# Co-occurrence Pattern of Posttraumatic Stress Disorder and Depression in People Living With HIV: A Latent Profile Analysis

**DOI:** 10.3389/fpsyg.2021.666766

**Published:** 2021-05-05

**Authors:** Jingjing Meng, Chulei Tang, Xueling Xiao, Maritta Välimäki, Honghong Wang

**Affiliations:** ^1^Xiangya Nursing School of Central South University, Changsha, China; ^2^Department of Nursing Science, University of Turku, Turku, Finland

**Keywords:** posttraumatic stress disorder, depression, comorbidity, people living with HIV, latent profile analysis

## Abstract

**Background**: The comorbidity of posttraumatic stress disorder (PTSD) and depression is common among people living with the HIV (PLWH). Given the high prevalence and serious clinical consequences of the comorbidity of these two disorders, we conducted a latent profile analysis to examine the co-occurrence pattern of PTSD and depression in PLWH.

**Methods**: The data for this cross-sectional study of PLWH were collected from 602 patients with HIV in China. A secondary analysis using latent profile analysis was conducted to examine HIV-related PTSD and depression symptoms.

**Results:** A four-class solution fits the data best, with the four classes characterized as asymptomatic (42.9%), mild symptoms (33.9%), low to moderate symptoms (19.8%), and high to moderate symptoms (3.4%). The severity of PTSD and depression symptoms was comparable in this solution, and no group was dominated by PTSD or depression.

**Conclusion**: The absence of a distinct subcluster of PLWH with only PTSD or depression symptoms supports that PTSD and depression in PLWH are psychopathological manifestations after traumatic exposures. Health care staff should pay more attention to the existence of comorbid symptoms of individuals, develop integrated interventions for the symptoms cluster, and evaluate their effectiveness in clinical practice.

## Introduction

Symptoms of posttraumatic stress disorder (PTSD) and depression are common among people living with the HIV (PLWH). The global prevalence of PTSD in PLWH ranges from 5 to 74% (Sherr et al., 2011) and depression averages 31% (Rezaei et al., 2019) in this population. PTSD and depression typically co-occur after a traumatic event (Bonde et al., 2016). It has been shown that 52% (*N* = 6,670) of individuals with PTSD have co-occurring major depressive disorder (Rytwinski et al., 2013). Some studies have found that PLWH who reported symptoms that meet the diagnostic criteria for PTSD and depression comorbidities were less likely to adhere to antiretroviral therapy and were more likely to have a detected viral load and a low rate of CD4 cells, which means accelerated disease progression (Boarts et al., 2006; Ebrahimzadeh et al., 2019). In view of the high prevalence and serious clinical consequences of PTSD and depression comorbidity, there is an urgent need to further understand the relationship between PTSD and depression.

The etiology-based hypothesis is the most classic explanation for the relationship between PTSD and depression (Stander et al., 2014), while the causal hypothesis posits that PTSD is a causal risk factor for depression (Ginzburg et al., 2010; Park et al., 2012) or, vice versa, that depression is a causal risk factor for PTSD (Arbisi et al., 2012; Goodwin et al., 2012). In contrast, the common factor hypothesis believes that PTSD and depression are independent outcomes of a common set of risk factors or vulnerabilities, such as military exposure (Hoge et al., 2006; O’Toole et al., 2009), genetic risk (Koenen et al., 2008), and past psychiatric treatment (O’Donnell et al., 2004), rather than direct causality. This third hypothesis assumes that the relationship between PTSD and depression is coincidental, even illusory. As PTSD and depression share some nonspecific characteristics, such as sleep disorders, anorexia, and others, the comorbidity may be overestimated (Elhai and Palmieri, 2011). Consequently, some researchers have held that PTSD and depression are indistinguishable single forms of psychopathology after traumatic stress (O’Donnell et al., 2004; Norman et al., 2011), while other researchers have argued that they are two independent but correlated sequelae after trauma (Blanchard et al., 1998).

Regardless of previous hypotheses, research on PTSD and depression comorbidity in PLWH has mainly focused on the prevalence and its correlates (Sledjeski et al., 2005; Boarts et al., 2006; Vranceanu et al., 2008; Klis et al., 2011; Verhey et al., 2018). In these cases, the assessment of comorbidities has solely been based on categorical diagnoses that divide individuals into negative and positive cases without considering clinically significant subthreshold symptoms; this might further lead to erroneous conclusions about the relationship between PTSD and depression.

A few studies have used factor analysis to characterize the latent structure of PTSD and depression. Elhai et al. (2011) found that the nonspecific dimension of PTSD that is essentially associated with depression is dysphoria. Other factor analysis studies have reported that PTSD and depression are loads on the anxious-misery factor (Cox et al., 2002; Miller et al., 2008) or distress factor (Slade and Watson, 2006). However, factor analysis may ignore the differences in comorbidity symptoms among heterogeneous individuals. It is also unclear whether the relationship between PTSD and depression varies depending on the type of trauma. We have not found studies that have investigated both PTSD and depression among the Chinese PLWH population. Therefore, understanding the co-occurrence pattern of these two disorders in this population would contribute to preventing and treating mental health problems.

Latent class analysis and latent profile analysis are person-centered statistical procedures that can be used to classify individuals into homogeneous subgroups based on shared symptom patterns (McCutcheon, 1987). Latent class analysis uses categorical indicators, while latent profile analysis uses continuous indicators, and both are based on the range of all symptoms rather than relying on categorical diagnosis. In addition, the continuous indicators used by latent profile analysis can reflect the severity of the symptoms rather than only reflect on the presence or absence of each symptom as dichotomous indicators. Latent profile analysis transcends the limitations of using factor analysis and diagnostic classification and may, therefore, contribute to a better understanding of the relationship between PTSD and depression. A few recent studies have used latent class or profile analysis to explore the relationship between PTSD and depression, but the results were inconsistent. Some studies have shown that the symptom severity of PTSD and depression is parallel in homogeneous subgroups (Au et al., 2013; Hruska et al., 2014; Armour et al., 2015), while others have shown that subgroups have PTSD or depression as the dominant symptom (Cao et al., 2015; Contractor et al., 2017). To our knowledge, neither latent class nor profile analysis has been used to examine the comorbidity pattern of PTSD and depression in PLWH.

It is possible that unclear diagnostic criteria and poor differential diagnosis led to the assumption of a comorbidity of PTSD and depression (Simms et al., 2002; Spitzer et al., 2007). Many studies (Tsai et al., 2015; Fung et al., 2019) have diagnosed PTSD based on the Diagnostic and Statistical Manual of Mental Disorders, fifth edition (DSM-V; American Psychiatric Association, 2013), which revised and refined the diagnostic criteria for PTSD to extend the symptom structure from three subgroups to four subgroups, including re-experience, avoidance, negative cognitions and mood, and arousal symptoms. Compared to the DSM-IV, the DSM-V subgroups ruled out numbing symptoms, and avoidance symptoms became more limited. In addition, the new symptoms contained in the DSM-V may not be specific to PTSD; for example, negative expectation is more closely related to depression (Koffel et al., 2012). Thus, the newly proposed symptoms might degrade the differential diagnosis and affect comorbidity prevalence rates (Friedman et al., 2011).

To better understand the relationship between specific PTSD subclusters and depression symptoms, we analyzed PTSD symptoms based on the DSM-IV diagnosis. Accounting for this, we investigated the co-occurring pattern between PTSD and depression using data collected from a sample of PLWH in China. Our study aims were 2-fold: (1) to identify the optimal latent class solution in categorizing participants based on their PTSD and depression symptom profiles and (2) to explain the characteristics of the optimal latent class solution.

## Materials and Methods

### Setting and Participants

This is the secondary analysis of the cross-sectional data from a study evaluating the efficacy of a resilience-informed trauma treatment in PLWH. Participants were recruited from a comprehensive hospital in Hunan Province, where they were treated between September 30, 2018, and January 1, 2019. The hospital was selected because it represents a typical tertiary comprehensive hospital in its area. The hospital includes a total of 63 clinical specialties and more than 1,500 beds, with AIDS being the provincial clinical key specialty. The hospital itself is the designated treatment institution for AIDS patients in the survey area.

The study population included those patients who were admitted to the study clinics or wards during the data collection period and fulfilled the following eligibility criteria: (1) had been diagnosed with HIV and registered with the Chinese Center for Disease Control and Prevention for more than a month (2) were age 18 years and older, and (3) were willing to join the study on a voluntary basis. Individuals who had been assessed by psychiatrists as having cognitive impairment were excluded from the study.

### Procedure

The study protocol was approved by the institutional review board of Central South University in Hunan (the institutional review board approval number: 2018040). Permission to collect the data in the hospital was approved by the competent authority.

Our survey used a consecutive sampling method and was conducted in the outpatient clinic or ward. All patients participated voluntarily, thus ensuring a high response rate for the participants. The trained investigators (AIDS specialists, psychotherapists, and master’s or doctoral students in nursing) explained the purpose and significance of the study, rights to withdraw, refusal, and anonymity to the participants before the investigation. If the participants were willing to join the study, they gave their verbal consent. Each eligible participant completed the questionnaire at the clinic/ward. For those with low education levels or who could not fill out the questionnaire by themselves, the investigator explained the meaning of each item and assisted them in completing it. Data collection for each participant lasted 20–35 min, and each participant received a reward of 50 yuan (US $8.3) after they completed the questionnaire and completeness of the items were checked. If any missing items were found, we asked the participant to fill them in if he or she wanted. In total, 602 participants voluntarily participated in the survey and filled out the questionnaire; no data were excluded.

### Measures

Assessed demographic characteristics included sex, age, marital status, educational level, employment, and monthly income.

HIV-related PTSD was assessed using the Chinese version of the PTSD Checklist-Civilian Version (PCL-C; Weathers et al., 1993; Su et al., 2011). The PCL-C is a 17-item scale based on the PTSD diagnostic criteria defined in the DSM-IV and has been widely used to capture PTSD symptoms associated with various traumatic events (Elhai et al., 2005; Li et al., 2010). The instrument, which has been tested in PLWH and proven to be acceptable for internal consistency, includes three clusters of characteristic symptoms of PTSD: (1) re-experiencing (e.g., “repeated, disturbing memories, thoughts, or images of the stressful experience”) (2) avoidance (e.g., “avoiding thinking, talking, or having feelings about the stressful experience”), and (3) hyperarousal (e.g., “trouble remembering important parts of the stressful experience”; Rubin et al., 2016; Kemppainen et al., 2017).

Participants were asked to rate how much they had been bothered by assessed symptoms during the past month based on the scale items (1 = not at all; 2 = a little bit; 3 = moderately; 4 = quite a bit; and 5 = extremely), such as “Repeated, disturbing dreams of a stressful experience from the past?” The scores of each item were summed to get a total score from 17 to 85, with higher scores indicating higher levels of PTSD symptoms. Suspicion of PTSD requires suffering at least one clinically significant symptom of re-experiencing, three for avoidance, and two for hyperarousal (a score of 3 or higher for each item; Weathers et al., 1993). Total PCL-C scores were calculated along with subscores according to the three-factor model of PTSD (re-experiencing, avoidance, and arousal). Cronbach’s alpha was 0.937 for the total, and 0.883, 0.871, and 0.843 for the re-experiencing, avoidance, and arousal subclusters, respectively.

Depression severity was assessed using the Self-Rating Depression Scale (SDS; Zung, 1965), which has been widely used and validated in China (Lee et al., 1994; Zeng et al., 2015). The SDS consists of 20 items rated 1–4 (1 = never or rarely, 2 = sometimes, 3 = most of the time, or 4 = almost all the time). Participants were asked to rate how they really felt regarding the assessed items over the previous week, such as “I have trouble sleeping at trouble.” Of the 20 items, 10 were expressed as negative experiences, such as “I sleep badly at night,” and 10 were expressed as positive experiences, such as “I feel hopeful about the future,” and were reverse scored. The total score ranged from 20 to 80. The standardized score was the original total score plus 1.25; less than 50 is considered normal, 50–59 mild depression, 60–69 moderate depression, and 70 or more severe depression. In the current sample, the scale’s Cronbach’s alpha was 0.870.

### Statistical Analysis

First, the sum scores of the SDS and the three PCL subclusters (re-experiencing, avoidance, and hyperarousal) were calculated. We carried out latent profile analysis for these four sum scores, not for the individual items, to minimize the number of indicators and promote model convergence while maximizing the interpretability of different solutions. Given the differences between the PCL and SDS scales, we converted these four sum scores to T scores for analysis.

Latent profile analysis was conducted using Mplus 7.4 software. We used a maximum likelihood estimation with robust standard errors to identify the participants’ classifications. To increase the chances that the analysis program would find the optimal solution with the highest log likelihood value, we used a sufficient number of 500 random sets of starting values and chose a sufficient number of iterations for the initial stage of the optimization (Uebersax, 2000). To determine the optimal number of classes, one- to five-class solutions were evaluated and compared based on fit indices, parsimony, and interpretability. To ensure the accuracy of the classification, each category would need at least 50 subjects, so the recommended minimum sample size was 250, and thus, the current size of 602 was considered sufficient (Yang, 2006; Nylund et al., 2007). We used information criteria (IC), including Bayesian information criterion (BIC), sample-size-adjusted Bayesian information criterion (aBIC), and Akaike information criterion (AIC), for model comparisons. In a series of models, the model with the smallest BIC, aBIC, or AIC value is preferred. In addition, the bootstrap likelihood ratio test (BLRT), entropy, and the Vuong-Lo-Mendell-Rubin (VLMR) likelihood ratio test and its adjusted version, the adjusted Lo-Mendell-Rubin (aLMR) likelihood ratio test were used to evaluate model fit. A significant BLRT, which is one of the most effective indices for accurately identifying the number of classes, suggests that k classes are a better model fit than k−1 classes (Nylund et al., 2007). Similarly, significant values for the VLMR and its adjusted version show that the estimated model fits significantly better than the model with one class less. Entropy, which ranges from 0 to 1, is an indicator that reflects the classification accuracy of the model. Values close to 1 indicate high classification accuracy, whereas values close to 0 indicate low classification certainty.

Once the optimal class solution was determined, we conducted the subsequent analysis based on the class assignment using SPSS 18.0. We used a series of ANOVAs to examine the differences between latent classes in depression (SDS total scores) and PTSD (total scores and three-subgroup scores). All significant ANOVAs were followed up with *post-hoc* pairwise comparisons using Dunnett’s T3, which does not assume an equal variance. We also performed a chi-square test to examine the differences in the prevalence of PTSD and the severity of depression between classes. A value of *p* ≤ 0.05 was considered statistically significant for all analyses.

## Results

### Descriptive Statistics

A total of 602 samples were included. The participants were mainly males (*N* = 536; 89.0%). The mean age of the sample was 33.81 years (*SD* = 11.36). Most participants (*N* = 430; 71.4%) were unmarried or currently separated from their partner. More than half (*N* = 342; 56.8%) had a college education or above. The median individual monthly income was about 4,000–5,000 yuan (US $571–$714), and most were employed (*N* = 437; 72.6%). About two-thirds (*N* = 473, 78.6%) of participants had been diagnosed with HIV for more than 3 months.

The means for the PCL-C total scores (along with those for the three dimensions) and SDS total scores were as follows: PCL-C total, *M* = 34.54, *SD* = 13.58; re-experiencing, *M* = 10.23, *SD* = 4.60; avoidance, *M* = 14.49, *SD* = 6.03; hyperarousal, *M* = 9.82, *SD* = 4.42; and SDS total, *M* = 48.24, *SD* = 12.64. According to the criteria requiring at least one re-experiencing symptom, three avoidance symptoms, and two hyperarousal symptoms, the prevalence of probable PTSD was 26.2% (*N* = 158). Based on a cutoff score of 50, 45.7% (*N* = 275) of participants reported depressive symptoms, of which 25.6% (*N* = 154) were mild, 16.1% (*N* = 97) were moderate, and 4.0% (*N* = 24) were severe. The comorbidity rate of PTSD and depression was 19.6% (*N* = 118).

### Identification of Latent Classes

**Figure 1:**
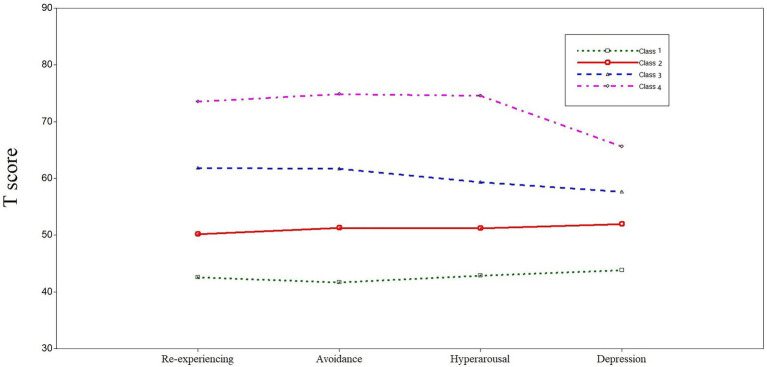
Posttraumatic stress disorder (PTSD) Checklist-Civilian Version (PCL-C) and Self-Rating Depression Scale (SDS) standardized subscores for the four-class model of symptom profiles.

Fit indices for one- to five-class solutions for the latent profile analysis models are presented in Table 1, and the four-class solution yielded the best fit for the data. The BIC, aBIC, and AIC values for the four-class solution were lower than those of the previous three solutions. Although the subsequent five-class solution showed lower IC values, the decreases in the fit indices were smaller, which meant IC values were flattening (DiStefano and Kamphaus, 2006). In the five-class solution, the value of *p* associated with both the VLMR and aLMR (*p* = 0.4004 and *p* = 0.4079, respectively) indicated that the five-class model did not fit significantly better than the more parsimonious four-class solution (Nylund et al., 2007). While significant BLRT values and close-to-1 entropy were found across solutions, the four-class solution was preferred to subsequent solutions for parsimony. Based on the principle of interpretability, the indicators of the four-class solution were better differentiated than those for the five-class solution (Figure 1). Average latent class assignment probabilities for individuals assigned to each class were 0.872, 0.985, 0.933, and 0.949, respectively, which indicated a high precision and reliability of the classification.

**Table 1:** Fit indices for latent profile analyses.

	BIC	aBIC	AIC	BLRT *p*	Entropy	aLMR *p*	VLMR *p*
One class	17889.309	17863.911	17854.107	-	-	-	-
Two classes	16920.616	16879.344	16863.413	0.0000	0.887	0.0000	0.0000
Three classes	16678.351	16621.205	16599.146	0.0000	0.830	0.1818	0.1753
**Four classes**	**16510.241**	**16437.222**	**16409.035**	**0.0000**	**0.864**	**0.0001**	**0.0000**
Five classes	16451.789	16362.896	16328.581	0.0000	0.868	0.4079	0.4004

Bold indicates best fit. Entropy and value of p for BLRT, aLMR, and VLMR are not applicable to a one-class solution. BIC, Bayesian information criterion; aBIC, sample-size-adjusted Bayesian information criterion; AIC, Akaike information criterion; BLRT, bootstrap likelihood ratio test; aLMR, adjusted Lo-Mendell-Rubin; VLMR, Vuong-Lo-Mendell-Rubin.

### Characteristics of Latent Classes

The severity of PTSD and depression gradually increased from Class 1 to Class 4 (Figure 1). Classes were defined as asymptomatic (Class 1), mild symptoms (Class 2), low-moderate symptoms (Class 3), and high-moderate symptoms (Class 4). The severity of PTSD symptoms and depression symptoms in the four classes were comparable, and no group was dominated by PTSD or depression.

All classes except Class 4 (*N* = 21, 3.4%) contained more than 50 individuals. ANOVA results showed that there were significant differences in SDS scores, PCL total scores, and the three individual PCL subgroup scores among the different classes. *Post-hoc* pairwise comparisons showed the severity of symptoms of each class and the accuracy of class classification. All four classes were significantly differentiated from each other in the symptom severity of PTSD and depression. Table 2 shows the proportion of individuals assigned to each class, the mean and SD of the unstandardized PCL and SDS, and the results of the ANOVA and the *post-hoc* pairwise comparisons. The chi-square test showed that there were significant differences in PTSD prevalence and depression severity among the four classes (Table 3).

**Table 2:** Unstandardized class means for four-class model.

	Class 1 *M*(*SD*) *N* = 258 (42.9%)	Class 2 *M*(*SD*) *N* = 204 (33.9%)	Class 3 *M*(*SD*) *N* = 119 (19.8%)	Class 4 *M*(*SD*) *N* = 21 (3.4%)	Class comparisons *F* (*df* = 3)	*p*	*Post-hoc* pairwise comparisons
Re-experiencing	6.74 (1.64)	10.26 (2.52)	15.78 (3.17)	21.23 (3.06)	555.05	<0.001	4 > 3 > 2 > 1
Avoidance	9.37 (2.13)	15.26 (2.84)	21.59 (3.14)	29.62 (3.29)	842.24	<0.001	4 > 3 > 2 > 1
Hyperarousal	6.52 (1.75)	10.37 (3.00)	14.13 (3.35)	20.76 (3.33)	363.92	<0.001	4 > 3 > 2 > 1
PCL (total)	22.63 (3.75)	35.89 (4.37)	51.50 (4.53)	71.62 (6.30)	1866.16	<0.001	4 > 3 > 2 > 1
SDS (depression)	39.54 (9.23)	51.27 (9.41)	58.19 (9.83)	69.29 (10.49)	158.33	<0.001	4 > 3 > 2 > 1

PCL, posttraumatic stress disorder Checklist-Civilian Version; SDS, Self-Rating Depression Scale.

**Table 3:** Difference in symptom severity among classes by chi-square test.

	Class 1 *N* (%)	Class 2 *N* (%)	Class 3 *N* (%)	Class 4 *N* (%)	*X*^2^ (*df* = 3)	*p*
Probable PTSD						
Positive	0 (0)	39 (19.1)	98 (82.4)	21 (100)	349.70	<0.001
Negative	258 (100)	165 (80.9)	21 (17.6)	0		
Depression severity						
Normal	214 (82.9)	88 (43.1)	24 (20.2)	1 (4.8)	280.56	<0.001
Mild	36 (14.0)	76 (37.3)	41 (34.5)	1 (4.8)		
Moderate	8 (3.1)	36 (17.6)	43 (36.1)	10 (47.6)		
Severe	0	4 (2.0)	11 (9.2)	9 (42.8)		

Most individuals in Class 1 (82.9%) had normal depression levels, and although some showed mild PTSD symptoms, none were diagnosed with probable PTSD. Individuals in Class 2 reported mild PTSD symptoms and depression, with nearly one in five individuals diagnosed with probable PTSD. Individuals in Class 3 reported moderate PTSD symptoms and depression. Although their level of depression was higher than that in Class 2, the average level was still mild but extremely close to the level of moderate depression, according to the cutoff scores. Individuals in Class 4 reported the highest severity of symptoms. All of this class had severe PTSD symptoms and were diagnosed as probable PTSD. Moderate or severe depression was reported in 90.4% of Class 4. Their average depression level was the highest among the four classes and was in the boundary of moderate and severe.

## Discussion

To our best knowledge, the present study is the first to use latent profile analysis to describe the co-occurring pattern of PTSD and depression in PLWH. Based on the data, we found four parallel profiles, including asymptomatic, mild symptoms, low-moderate symptoms, and high-moderate symptoms. The symptom severity among the profiles was comparable, and no group was dominated by PTSD subclusters or depression symptoms. These findings fill in the gap of using latent profile analysis to examine HIV-related PTSD and depression comorbidity patterns in PLWH, and they reveal the indistinctiveness between PTSD subclusters and depression.

Our results indicated that PTSD and depression were very common in the study population, supporting previous findings of a high incidence of psychopathology in patients with HIV (Beckford Jarrett et al., 2018; Ebrahimzadeh et al., 2019). We also found a high co-occurrence of these two disorders. PLWH experience traumatic or stressful life events following HIV diagnosis, including HIV physical symptoms, lower satisfaction with social support, and HIV-related stigma and discrimination (Katz and Nevid, 2005; Andu et al., 2018), all of which increase the probability of PTSD and depression comorbidity. Previous studies have also shown that regular treatment can cause emotional distress in PLWH (Theuninck et al., 2010). Our samples all received regular antiretroviral therapy at the study site. The distress might be caused by the physical discomfort of the treatment and the negative impact of the treatment on the patient’s occupation and social aspects.

Previous studies have found that the severity of PTSD and depressive symptoms were tightly correlated and that no subgroup with comorbidity patterns were dominated by PTSD or depression (Norman et al., 2011; Au et al., 2013; Armour et al., 2015). We also found similar parallel classes. The vast majority of participants were diagnosed with HIV infection for at least 3 months, which may have caused the distinction between PTSD and depression to be less obvious as the symptoms gradually become chronic. Similarly, the research of O’Donnell et al. (2004) suggested that depression was distinct from PTSD in the early months post trauma, but over time, the distinction between these symptoms became less obvious. A few studies have found a subgroup of individuals with either PTSD or depression to be dominant (Ikin et al., 2010; Cao et al., 2015), and this discrepancy may be caused by the differences in post-traumatic time points and trauma types.

It has been proposed that the diagnostic criteria of PTSD in the DSM-V include dimensions that overlap with other emotional disorders, such as depression, which may reduce the distinction between PTSD and depression or other disorders (Gootzeit and Markon, 2011). Thus, to explore the distinction between PTSD and depression, we used the diagnostic criteria for PTSD defined in the DSM-IV without expanding the symptom structure. The current parallel PTSD and depression results show that even the DSM-IV cannot distinguish PTSD from co-occurring depressive symptoms, which proves the indistinction of PTSD and depressive symptoms and suggests that PTSD and depression may be comprehensive psychopathological manifestations after trauma. A study using the DSM-V to define PTSD found that PTSD and depression represented distinct constructs when PTSD was at a higher level of severity (Contractor et al., 2017). More than half of the participants in our study had mild or moderate PTSD symptoms, and the difference in the severity of the symptoms might explain the inconsistency with the results of previous studies.

Another noteworthy finding in this study was that all three PTSD subclusters and depression could be well-differentiated among the four classes, as characterized by the parallel varying severity of symptoms, which was in line with findings of previous studies using latent class or profile analysis to examine the association between PTSD and depression (Au et al., 2013; Contractor et al., 2017). Judging from the diagnostic rates across classes, the proportion of individuals in each who met the diagnostic criteria of probable PTSD and depression increased through Class 1 to Class 4. Dividing individuals directly into negative and positive according to diagnostic criteria would ignore subthreshold symptoms and would not identify heterogeneous individuals (McCutcheon, 1987). Our findings confirmed that the individuals’ comorbid symptoms could be quantitatively and qualitatively differentiated better according to the severity of the symptoms, suggesting that future studies can design personalized treatment plans based on the severity of individual symptoms.

There are several limitations to this study. First, our study was a cross-sectional survey, which cannot evaluate the stability of latent profiles over time through multi-level modeling. Future research should focus on different post-traumatic time points and use latent transition analysis on longitudinal data to empirically examine changes in latent profiles. Second, the current findings might be limited by specific types of HIV-related trauma, thus affecting the generalizability in other types of trauma. Third, we did not assess for other possible factors, such as rumination (Dell’Osso et al., 2019) and social support (Adams et al., 2019), which may affect the comorbidity pattern of PTSD and depression. Future studies need to consider more risk factors when describing the relationship between these two disorders. Fourth, only PTSD, not depression, was assessed using the DSM-IV criteria in this study. Therefore, replications using DSM-IV criteria for PTSD and depression are also needed. Finally, our measurement tools cannot diagnose PTSD and depression clinically. Future research should use assessment tools and structured interviews for formal clinical diagnosis.

Despite these limitations, this is the first study using latent profile analysis to explore the relationship between HIV-related PTSD and depression. We used dimensional indicators rather than only categorical diagnoses to reflect the full range of comorbid symptoms’ severity among subclasses in PLWH. Our sample was representative considering the consecutive sampling method and relatively large sample size, which increased the generalizability and reliability of the classification of heterogeneous individuals in PLWH. In addition, the finding of comorbidity patterns of PTSD and depression in PLWH has great therapeutic significance. At present, PTSD and depression interventions for HIV-positive people in China and even globally are usually conducted separately (Himelhoch et al., 2013; Ye et al., 2018). Treatment focused on PTSD may neglect subthreshold depression symptoms and vice versa. To develop more targeted and integrated interventions, future research needs to explore the symptom clusters with a sensitive response to treatment to further develop and evaluate the treatment effect for comorbid PTSD and depression. Considering that the severity of PTSD and depression comorbid symptoms is critical to identifying potential subgroups, both clinicians and patients may benefit from using a transdiagnostic treatment regimen targeting negative effects for PLWH with the co-occuring symptoms.

## Conclusion

The current study suggests that PTSD and depression in PLWH are possibly psychopathological manifestations after HIV diagnosis. More studies are needed in the future to verify this conclusion under different types of trauma. In addition, researchers and health care staff should pay more attention to the existence of comorbid symptoms, develop integrated interventions for the symptoms cluster, and evaluate their effectiveness.

## Data Availability Statement

The raw data supporting the conclusions of this article will be made available by the authors, without undue reservation.

## Ethics Statement

The studies involving human participants were reviewed and approved by the Institutional Review Board of Behavioral and Nursing Research in School of Nursing of Central South University in Hunan, China. Written informed consent for participation was not required for this study in accordance with the national legislation and the institutional requirements.

## Author Contributions

JM conducted the background research, designed and performed the statistical analyses, and wrote the first draft of the manuscript. CT and XX conceptualized the study design, conducted the study, assisted with the analysis, and commented on drafts. MV supervised the analyses and provided critical revision to the manuscript. HW revised the first draft of the manuscript and commented on drafts. All authors contributed to the article and approved the submitted version.

### Conflict of Interest

The authors declare that the research was conducted in the absence of any commercial or financial relationships that could be construed as a potential conflict of interest.
